# A Lack of Premature Termination Codon Read-Through Efficacy of PTC124 (Ataluren) in a Diverse Array of Reporter Assays

**DOI:** 10.1371/journal.pbio.1001593

**Published:** 2013-06-25

**Authors:** Stuart P. McElroy, Toshifumi Nomura, Leah S. Torrie, Emma Warbrick, Ulrike Gartner, Gavin Wood, W. H. Irwin McLean

**Affiliations:** 1 Drug Discovery Unit, Division of Biological Chemistry and Drug Discovery, College of Life Sciences, James Black Centre, Dow Street, University of Dundee, Dundee, United Kingdom; 2 Department of Dermatology, Hokkaido University Graduate School of Medicine, Sapporo, Japan; 3 Division of Molecular Medicine, College of Life Sciences, Medical Sciences Institute, Dow Street, University of Dundee, Dundee, United Kingdom; Brandeis University, United States of America

## Abstract

The drug molecule PTC124 (Ataluren) has been described as a read-through agent, capable of suppressing premature termination codons (PTCs) and restoring functional protein production from genes disrupted by nonsense mutations. Following the discovery of PTC124 there was some controversy regarding its mechanism of action with two reports attributing its activity to an off-target effect on the Firefly luciferase (FLuc) reporter used in the development of the molecule. Despite questions remaining as to its mechanism of action, development of PTC124 continued into the clinic and it is being actively pursued as a potential nonsense mutation therapy. To thoroughly test the ability of PTC124 to read through nonsense mutations, we conducted a detailed assessment comparing the efficacy of PTC124 with the classical aminoglycoside antibiotic read-through agent geneticin (G418) across a diverse range of *in vitro* reporter assays. We can confirm the off-target FLuc activity of PTC124 but found that, while G418 exhibits varying activity in every read-through assay, there is no evidence of activity for PTC124.

## Abstract 2

Numerous nonsense mutation reporter assays fail to reveal read-through activity for the drug PTC124.

## Author Summary

Ten percent of all single-gene hereditary diseases are caused by nonsense mutations. These are alterations in the DNA sequence of a protein-coding gene that cause the ribosome to prematurely finish translating the gene transcript before a full-length, active protein can be produced. In 2007 a drug was developed called PTC124 (latterly known as Ataluren), which was reported to help the ribosome skip over the premature stop, restore production of functional protein, and thereby potentially treat these genetic diseases. In 2009, however, questions were raised about the initial discovery of this drug; PTC124 was shown to interfere with the assay used in its discovery in a way that might be mistaken for genuine activity. As doubts regarding PTC124's efficacy remain unresolved, here we conducted a thorough and systematic investigation of the proposed mechanism of action of PTC124 in a wide array of cell-based assays. We found no evidence of such translational read-through activity for PTC124, suggesting that its development may indeed have been a consequence of the choice of assay used in the drug discovery process.

## Introduction

Nonsense mutations are a type of genetic defect in which an amino acid codon is substituted by a TGA, TAG, or TAA stop codon, thereby interrupting the coding sequence of a protein-encoding gene. These mutations represent one major class of premature termination codon mutations (PTCs); out-of-frame insertion/deletion mutations can also lead to a PTC via a frameshift mechanism. Nonsense mutations prematurely terminate translation and result in production of either a truncated, non-functional protein, or, in many cases, pre-translational destruction of the transcript via nonsense-mediated mRNA decay [1]. PTC mutations are responsible for ∼10% of all human genetic disease and there is currently no available treatment [2]. As such, the discovery of the small molecule PTC124 provides hope for the development of a drug that can facilitate the read-through of PTCs and restore functional protein production [3]. Such a molecule would be applicable to a wide range of incurable hereditary diseases and some forms of cancer. The molecule was first described as effective in animal models of Duchenne muscular dystrophy (DMD) [3], and the developers subsequently reported improvements in protein production in models of cystic fibrosis (CF) [4] and dysferlin deficiency [5]. This led to human clinical trials, where improvements in chloride channel conductance have been reported in CF patients [6],[7],[8]. Despite this clinical success, there have been studies that cast doubt upon the underlying mechanism of action of PTC124 [9],[10].

Originally developed by optimising hit compounds identified following two high-throughput screening campaigns, the assay utilised in this effort was a cell-based firefly luciferase (FLuc) reporter containing an in-frame PTC, specifically the nonsense mutation TGA [3]. The authors describe the up-regulation of FLuc activity in response to PTC124 which they attribute to read-through of the PTC. However, it has subsequently been reported that the compound is a highly potent FLuc inhibitor, and the suggestion was made that this could be responsible for the up-regulation of luciferase signal that Welch *et al.* observed [9],[10]. The rationale for this counterintuitive effect has been reviewed in detail [11]. Briefly, cells transfected with a FLuc construct containing a PTC in the reading frame can still produce a very small but steady amount of full-length FLuc due to natural read-through of the ‘leaky’ PTC. Incubation of the cells with inhibitor results in the compound binding and stabilising the protein, reducing its proteolytic degradation, increasing its half-life, and increasing the effective concentration of active FLuc over the course of the incubation period. The last step of the experiment is to dilute the medium with lysis buffer and add a very high concentration of the FLuc substrate luciferin. The luciferin essentially out-competes the reversible inhibitor and produces a luminescent signal which is mistakenly attributed to facilitated read-through of the PTC mutation. The molecular basis of PTC124 inhibition of FLuc was described in detail by Auld *et al.* with the X-ray crystal structure showing the enzyme binding and converting PTC124 to a PTC124-AMP adduct which has extremely high affinity for the enzyme [10].

Whilst these two studies described the mechanism by which PTC124 can up-regulate FLuc activity independently of PTC read-through, they only cursorily addressed whether the compound possesses genuine read-through activity. They generated a structurally unrelated *Renilla* luciferase (RLuc) construct containing a PTC and, using transient transfection, observed no activity with PTC124 and a modest two-fold increase in activity with the aminoglycoside gentamicin [9]. This low assay sensitivity, the potential for a discrepancy between transient and stably transfected reporters, and the positive, albeit qualitative, read-through data generated with human and mouse models of DMD [3] were used to argue that PTC124 may exhibit genuine read-through activity in spite of its interference in the FLuc assay [12].

To address some of the uncertainty surrounding the efficacy of PTC124 in promoting read-through of PTCs, we compared its activity with the well-characterised read-through agent geneticin (G418) in multiple stably and transiently transfected PTC reporter cell lines. To be thorough, we used a diverse array of protein reporters and characterised compound activity across numerous PTC sequences.

## Results and Discussion

### PTC124 Increases FLuc Activity Independently of PTC Read-Through

**Figure 1:**
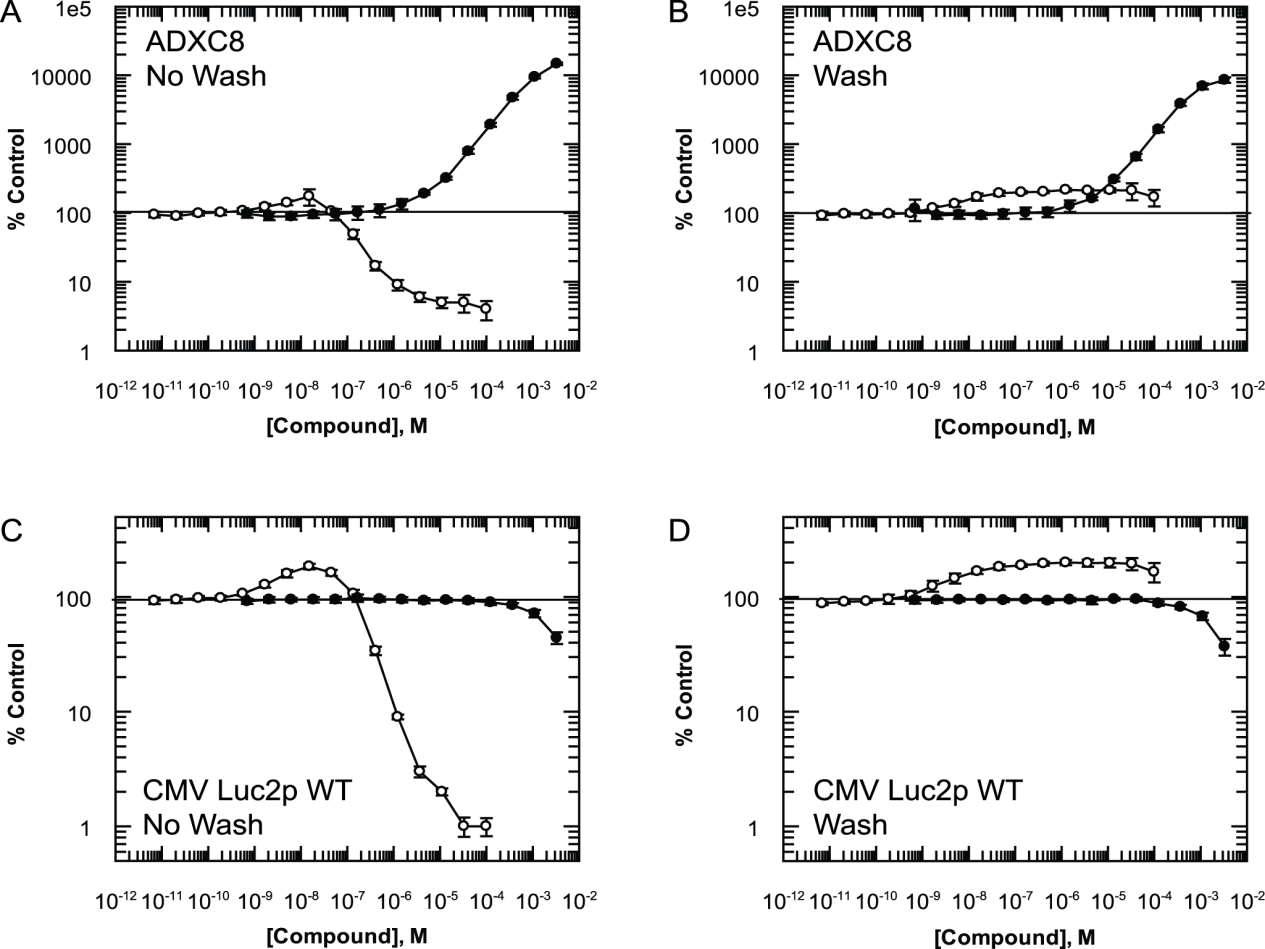
Differing activity of PTC124 and G418 in FLuc reporter cell lines. (A) Stop codon-containing FLuc activity in ADXC8 cells treated for 24 hours with G418 (closed circles) or PTC124 (open circles) which were not washed of compound prior to addition of luciferase detection buffer. (B) FLuc activity in ADXC8 cells in which compound was washed off prior to addition of detection buffer. Both compounds were also tested under the same conditions in WT-FLuc cells using the same (C) no wash and, (D) wash protocol. All data points represent mean ± standard deviation (n = 5).

We constructed a FLuc cell-based assay using AD293 (ADXC8) cells stably transfected with a FLuc2P construct containing an in-frame nonsense mutation (TGA) at position 223. The following (+4) base position is a G residue. For ease of understanding the relative position of this PTC in the FLuc reading frame and for all constructs used in this study, a graphical summary is included in Figure S1. Following 24-hour incubation with G418, FLuc activity in ADXC8 cells dose-dependently increased to a maximum at 3.3 mM of 14,800%±600% of DMSO-only control (Figure 1A, n = 5). This is in contrast to a maximum response of only 174%±46% of DMSO-only control (n = 5) with 0.15 µM PTC124. At concentrations higher than this, PTC124 dose-dependently inhibits FLuc activity, most likely due to the continued presence of compound when the lysis/detection (LD) buffer is added to the cells. To investigate if this inhibition is masking more pronounced stimulation of FLuc activity due to PTC read-through, compound was removed from cells by washing with media (three times, 10 minutes each) before adding the LD buffer. Under these conditions G418 still produced a pronounced dose-dependent increase in signal, albeit with a 43% reduction in the maximum response (8,500%±800% of DMSO-only control, Figure 1B, n = 5). The increase in signal caused by PTC124 was no longer inhibited at concentrations greater than 0.15 µM (171%±20% of DMSO-only control at 0.15 µM, n = 5 versus 170%±45% at 100 µM, n = 5). However, neither was the maximum response significantly greater than that observed in the presence of PTC124.

To investigate the effects of PTC124 on FLuc activity independently of the PTC, we stably transfected AD293 cells with a wild-type FLuc2P construct under control of the same promotor (CMV Luc2p WT). In this cell line, G418 did not affect FLuc activity, except for a slight reduction at 1 mM and above, most likely due to toxicity (Figure 1C). In contrast, PTC124-treated CMV Luc2P WT cells behaved in the same manner as observed for ADXC8 cells, with a maximum response of 185%±10% (n = 5) of control at 0.15 µM and marked inhibition at higher concentrations. Removal of compound prior to addition of LD buffer did not alter the lack of effect for G418-treated cells, but the PTC124-treated cells again exhibited the same profile of activity observed for ADXC8 cells (168%±9% at 0.15 µM, n = 5 versus 166%±32% at 100 µM, n = 5, Figure 1D).

These data show that the profile of PTC124 activity in two FLuc reporter assays is virtually identical whether or not a PTC is present. This is in agreement with Auld *et al.* that protein stabilisation is the mechanism through which PTC124 is increasing the FLuc signal. In contrast, the well-established read-through agent G418 produced a pronounced, dose-dependent increase in FLuc activity, orders of magnitude higher than that of PTC124 and had no effect in control cells. This positive control demonstrates that, for this cell type, *bona fide* read-through of PTCs increases protein levels to a greater extent than passive stabilisation from proteolytic degradation.

### G418 Increases Stably Transfected PTC-β-Galactosidase Enzyme Activity whilst PTC124 Has No Effect

**Figure 2:**
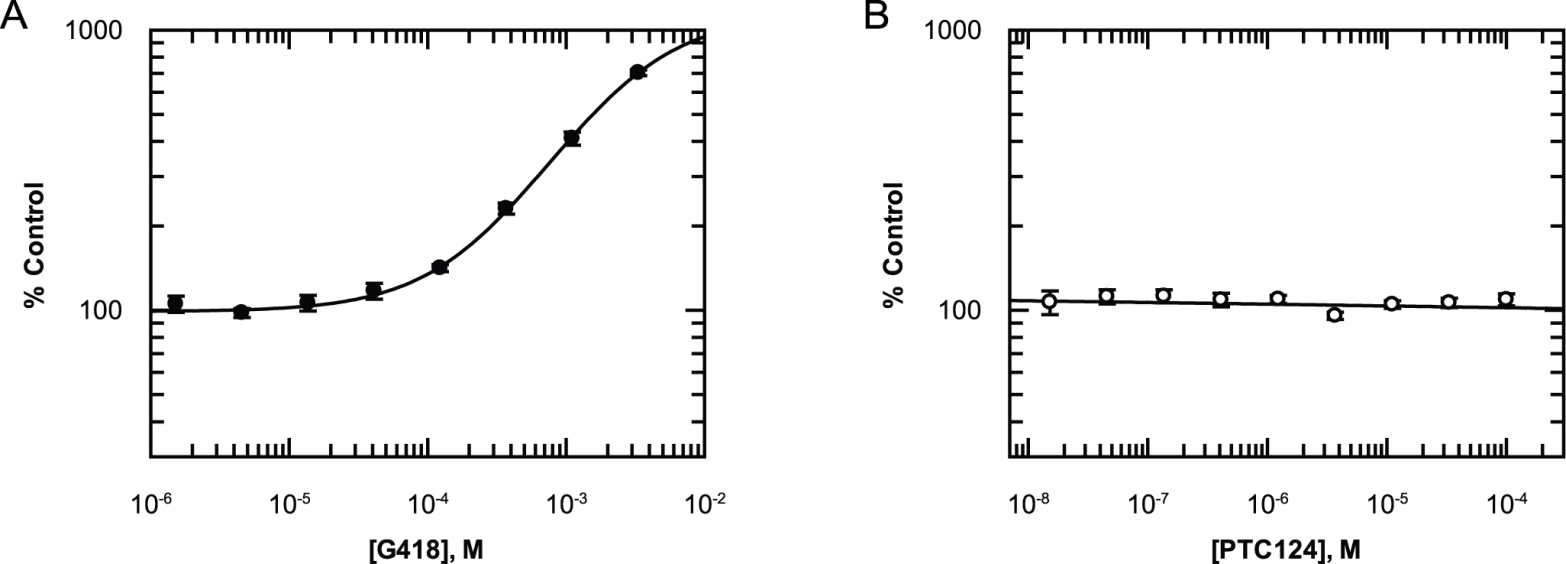
Lack of PTC124 efficacy with a stably expressed β-Galactosidase PTC-reporter. (A) Incubation with G418 for 24 hours leads to a dose-dependent increase in enzyme activity of stably transfected PTC-β-Gal. (B) 24-hour incubation with PTC124 at concentrations in excess of its reported effective concentration range of 0.1–30 µM has no effect. All data points represent mean ± standard deviation (n = 5).

To test for activity of PTC124 in a non-luciferase PTC reporter system we stably transfected AD293 cells with a β-galactosidase (β-Gal) construct containing an in-frame nonsense mutation (TGA-G) at position 320 within the *lac*Z gene (Figure S1). Incubation with G418 for 24 hours resulted in a dose-dependent increase in β-Gal activity (704%±16% at 3.3 mM, n = 5; Figure 2). PTC124 however had no effect at concentrations between 0.1 nM up to 100 µM. We intentionally did not test PTC124 at higher concentrations, as this is in excess of the published maximally effective *in vitro* and *in vivo* concentration range of between 0.1 and 30 µM [3],[4],[13]. Interestingly, the minimal effective G418 concentration required to increase β-Gal activity appears greater than that observed for the FLuc reporter, indicating that the different PTC reporter systems are differentially sensitive to read-through.

### G418 Increases Transiently Transfected PTC-RLuc Enzyme Activity whilst PTC124 Has No Effect

**Figure 3:**
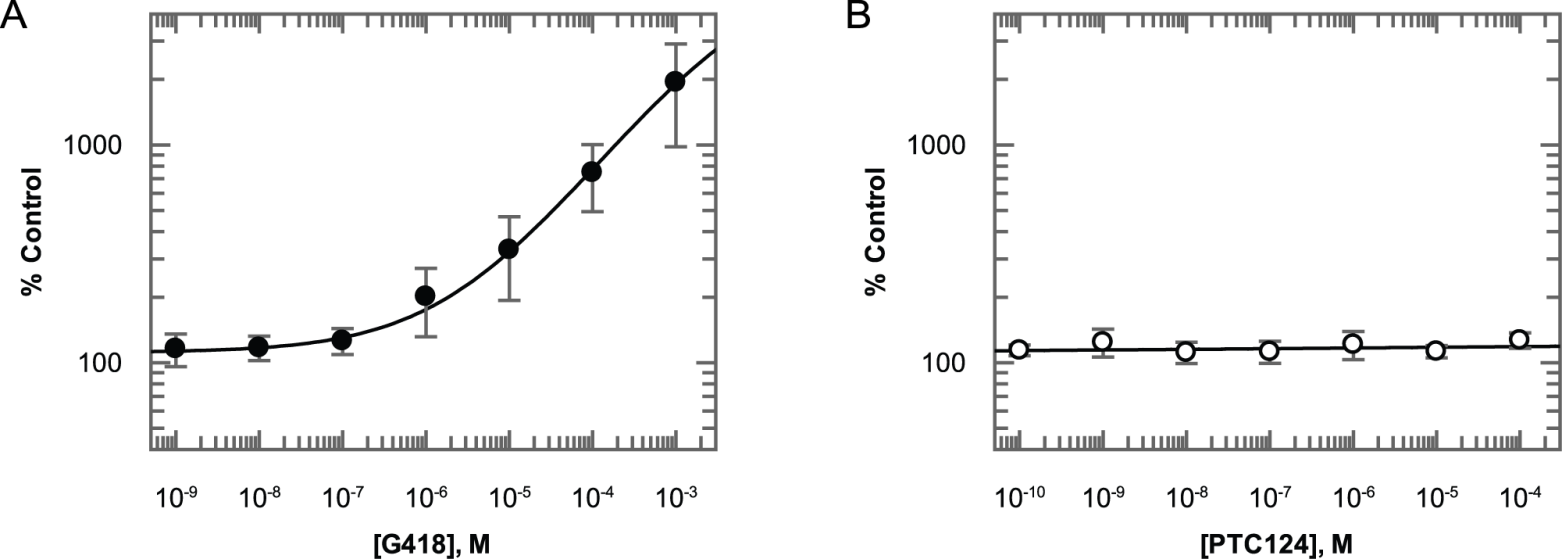
Lack of PTC124 efficacy with a transiently transfected RLuc PTC-reporter. (A) Incubation with G418 for 24 hours leads to a dose-dependent increase in enzyme activity of transiently transfected PTC-RLuc. (B) 24-hour incubation with PTC124 at concentrations in excess of its reported effective concentration range of 0.1–0 µM has no effect. All data points represent mean ± standard deviation (n = 5).

The reduced sensitivity of the β-Gal reporter to G418 may raise questions about the ability to accurately determine read-through with poorly efficacious compounds when switching between reporter assays. To ensure that a β-Gal assay–dependent shift in potency is not the reason for being unable to see activity with PTC124, a third PTC reporter was constructed using AD293 cells transiently transfected with a RLuc construct containing an in-frame nonsense mutation (TGA) at position 21 (Figure S1). Incubation with G418 for 24 hours resulted in a dose-dependent increase in RLuc activity with a minimal effective concentration similar to that observed with the FLuc reporter (Figure 3). In contrast, PTC124 had no effect at any concentration tested (Figure 3). This is consistent with the results from Auld *et al.* albeit with a different RLuc construct [9].

### G418 Increases Transiently Transfected Collagen VII Production in Two Independent PTC-Containing Constructs whilst PTC124 Has No Effect

**Figure 4:**
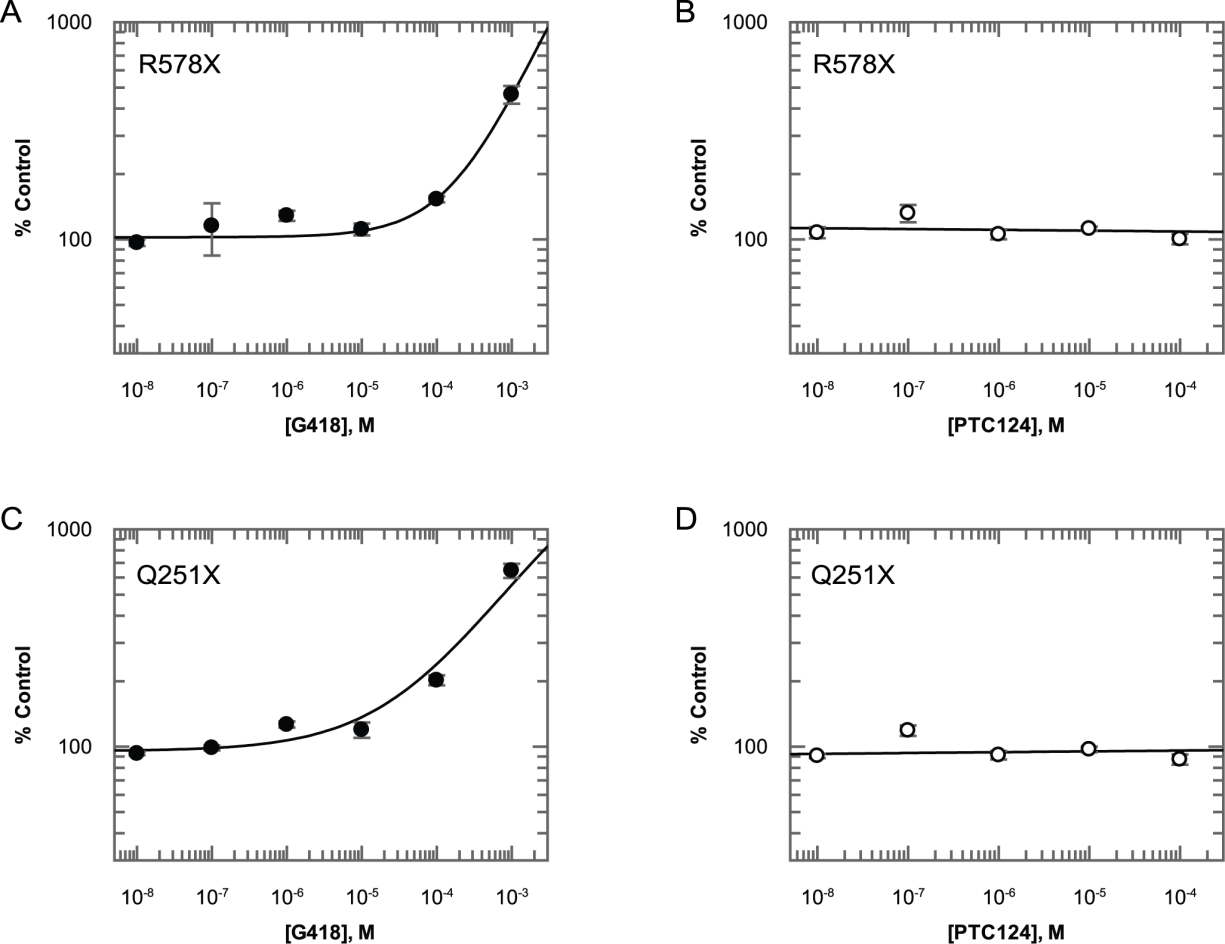
Lack of PTC124 efficacy with transiently transfected collagen VII PTC reporters. Dose-dependent increases in detectable protein with G418 but not PTC124 in R578X (A and B) and Q251X (C and D) PTC collagen VII constructs. All data points represent mean ± standard deviation (n = 4).

In all of the assays tested so far we used non-mammalian reporter constructs. To determine if PTC124 is active against full-length mammalian proteins, we generated two FLAG-tagged collagen VII constructs based upon the two main PTC mutations responsible for severe dystrophic epidermolysis bullosa in the human population, Q251X (TAG) and R578X (TGA) [14],[15] (Figure S1). For both constructs, transient transfection into AD293 cells and subsequent incubation with G418 resulted in detectable, dose-dependent increases in production of full-length protein as detected by ELISA, with maximal responses at 1 mM of 646%±48% of DMSO-only control (n = 4) and 464%±43% of DMSO only control (n = 4), respectively (Figure 4). Only collagen VII protein secreted into the medium is detected in this assay, which requires an N-terminal signal sequence. Further, capture onto the ELISA plate requires the presence of the C-terminal FLAG tag, and the detection antibody recognises the N-terminal NC1 domain, so only full-length protein will be detected (Figure S1). As was observed in all other PTC reporter assays, PTC124 did not increase protein production at any concentration tested.

### G418 Increases Protein Production for Every PTC Sequence but PTC124 Has No Effect

The termination efficiency of stop codons is heavily dependent upon their sequence (TGA, TAG, or TAA) and the nature of the nucleotide following the nonsense codon (+1 position) [16],[17],[18]. To determine if the sequence context has some influence over the lack of efficacy of PTC124 we have observed so far, we generated a number of Keratin 6a-YFP fusion constructs (K6a-YFP) with the cysteine codon at position 533 replaced by a TGA, TAG, or TAA and all variants of the base in the +1 position (Figure S1). YFP production was then determined by Western blotting using transiently transfected AD293 cells that were incubated with either compound or DMSO-only control for 24 hours. We selected a concentration of 200 µM for G418, which previous experiments suggest is tolerated by the cells but still elicits detectable read-through and 3 µM PTC124, which was described as the maximally effective *in vitro* concentration by Welch *et al.* [3].

**Figure 5:**
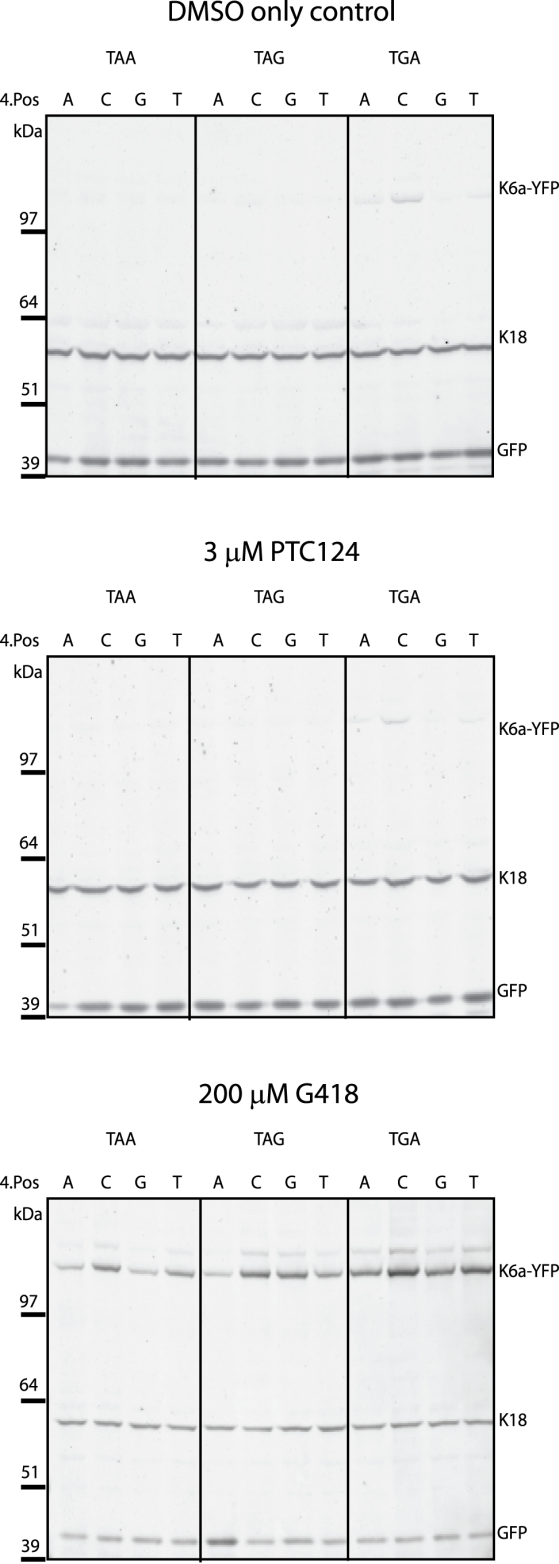
PTC124 does not increase detectable K6a-YFP production for any PTC sequence. Twelve PTC mutant K6a-YFP constructs were generated covering all potential PTC sequence contexts including variations in the +1 base. All constructs were transiently transfected into AD293 cells, which were subsequently treated with DMSO control, 200 µM G418, or 3 µM PTC124 for 24 hours before Western blots were conducted to determine presence of YFP, GFP transfection control, and K18 loading controls.

In DMSO-treated cells, K6a-YFP staining is predominantly very low level or undetectable for most PTC sequences (Figure 5). However, some PTCs are ‘leakier’ than others with a greater degree of basal protein production for TGA constructs, in particular TGAC. Exposure to G418 substantially increased K6a-YFP staining for all PTC sequences relative to DMSO-only controls (Figure 5). Again, the different PTC sequences appear to yield different degrees of read-through, with TAA being the most resistant (TAAA and TAAG in particular). In contrast, the effect of PTC124 was indistinguishable from the effect of DMSO alone on the expression of the various K6a-YFP constructs.

### Conclusions

We present an in-depth study of the comparative efficacy of two published read-through agents, PTC124 and G418. We used a diverse panel of PTC reporter assays, including transient transfection, stable cell lines, plate-based functional enzyme assays, and direct protein detection using ELISA and Western blotting. While we report activity with G418 in every assay, PTC124 exhibited no measurable effects. We also assessed the effects of sequence context upon read-through by testing 12 different constructs containing varying PTC sequences. In all cases G418 stimulated protein production, albeit to varying extent depending upon the sequence, whereas PTC124 had no effect.

All of these reporter assays utilised cDNA constructs. An argument may be made that cDNAs lack relevance due to the potential absence of mRNA regulatory processes that are present in endogenous genes, one of which could be the mechanism through which PTC124 exerts its read-through activity. Although this argument is valid, it would not be compatible with the discovery and development of PTC124, which was accomplished also using a cDNA construct.

While we and others find no evidence of read-through efficacy for PTC124 [9],[19],[20],[21],[22], there are independent studies detailing efficacy for PTC124 in non-FLuc read-through assays [13],[23],[24]. A greater understanding of this mechanistic discrepancy is very important for the future development of read-through drugs. Importantly, this should be accomplished by performing full and quantifiable dose–response studies of these drugs benchmarked against the activity of the aminoglycosides in multiple assays. Interestingly, the *in vitro*/*ex vivo* activity of PTC124 may be very different to that *in vivo*, as there are a number of reports for efficacy in *in vivo* models of disease and in clinical trials, in particular for CF [6],[7],[8]. In this regard a more thorough understanding of the mechanism of action of PTC124 would be highly beneficial.

## Materials and Methods

All cells were grown at 37°C and 5% CO_2_, in a humidified atmosphere. Cells were grown in DMEM supplemented with 10% v/v foetal calf serum (FCS). Cells were transfected using Lipofectamine 2000 (Invitrogen) according to the manufacturer's protocol. G418 was purchased from Gibco as 50 mg/ml stock solution in water. The PTC124 used for the majority of experiments was synthesised in the laboratory of Philip Cohen and was analysed using LCMS and NMR to confirm purity and molecular identity prior to usage (Figure S2, Figure S3). For the collagen VII and K6a-YFP assays, PTC124 was purchased from Exclusive Chemistry.

### FLuc Reporter Assays

The cytosine at positions 666 and 669 of the luc2P gene in pGL4.21[luc2P/puro] were both replaced with adenine using the QuickChange site-directed mutagenesis system (Stratagene) using the following primers: Luc2P-wtFLG.F 5′ CCG ATT CAG TCA TGC ACG AGA CCC CAT CTT CGG C 3′ and Luc2P-wtFLG.R 5′ GCC GAA GAT GGG GTC TCG TGC ATG ACT GAA TCG 3′. The codon for arginine 223 was subsequently replaced by a premature TGA stop codon using the following primers: Luc2P-TGA.F 5′ CCG ATT CAG TCA TGC ATG AGA CCC CAT CTT CGG C 3′ and Luc2P-TGA.R 5′ GCC GAA GAT GGG GTC TCA TGC ATG ACT GAA TCG 3′. A 0.9 kb BamH1/BglII fragment from pcDNA3 (Invitrogen) was subcloned into the mutated pGL4.21[luc2P/puro] plasmid so that the CMV promoter was inserted upstream of the luc2P coding sequence.

### Generating AD293 Cells That Stably Express Mutated FLuc (ADXC8 Cells)

AD293 cells were transfected with mutated pGL4.21[luc2P/puro]. The cells were placed under 1 µg/ml puromycin selection 24 hours post-transfection and grown until resistant foci were identified. Twenty-four puromycin-resistant clones were picked for further analysis. Cells were transfected with pRL-CMV (Promega) and treated with 600 µg/ml of gentamicin (Invitrogen). Cells were incubated for 24 hours and then FLuc/RLuc activity tested. Cells were washed with PBS and lysed with Passive Lysis buffer (Promega) for 15 minutes with shaking. Luciferase activity was detected with the Dual Luciferase Reporter System (Promega) according to the manufacturer's instructions. Luminescence was detected with a LUMIstar OPTIMA luminometer (BMG Labtech). The ratios of FLuc/RLuc activity were calculated and one clone was selected for further use.

To test for read-through resulting in FLuc activity, cells were seeded into white 96-well plates (Greiner) and incubated for 24 hours with G418 or PTC124 added to concentrations as shown in Figure 1. Luciferase activity was measured by monitoring luminescence on a TopCount luminometer (PerkinElmer) following the addition of 50 µl of lysis/detection buffer (comprising: 25 mM Tris-Phosphate, 8 mM MgCl_2_, 1 mM dithiothreitol, 0.5 mM ATP, 4 µM sodium pyrophosphate, 1% Triton X-100 (v/v), 0.5% (w/v) BSA, 15% glycerol (v/v), and 0.1 mg/ml luciferin).

### β-Gal Reporter Assays

The cysteine 320 codon of the *lac*Z gene in the pSV-β-galactosidase vector (Promega) was replaced by a premature TGA stop codon using the QuickChange site-directed mutagenesis system (Stratagene) with the following primers: pSV-*lac*Z-TGA.F 5′ GAT TGA AGC AGA AGC ATG AGA TGT CGG TTT CCG CG 3′ and pSV-*lac*Z-TGA.R 5′ CGC GGA AAC CGA CAT CGC AGG CTT CTG CTT CAA TC 3′. This reaction also replaced the cytosine at position 960 of the *lac*Z gene with adenine. A 3,738 bp BamH1/HindIII fragment containing the mutated *lac*Z construct was subcloned into the modified pGL4.21 vector described in ‘FLuc reporter assays’ (above) so that the mutated *lac*Z gene could be expressed from the CMV promoter.

### Generating AD293 Cells That Stably Express Mutated β-Gal

AD293 cells were transfected with the mutated pSV-β-galactosidase and grown under puromycin selection for one week. The cells were subsequently trypsinised and seeded at approximately one cell per well in 96-well plates. For characterisation of these clones, the cells were treated with 100 µg of geneticin (Invitrogen) for 24 hours, following which the cells were lysed with 1× Lysis Buffer (Promega) at room temperature for 1 hour with shaking at 900 rpm. 150 µl of assay buffer, consisting of 4 mg/ml CPRG (Roche), 4.5 M β-mercaptoethanol (Sigma-Aldrich), and 0.1 M magnesium chloride (Invitrogen) in 0.1 M phosphate buffer, pH 7.5, was added to each well. After 5 hours of incubation with the assay buffer, absorbance at 580 nm was detected using the EnVision (PerkinElmer), and the clone giving the strongest signal was selected for further use.

To test for read-through resulting in β-Gal activity, cells were seeded into white 96-well plates (Greiner) and incubated for 24 hours with G418 or PTC124 added to concentrations as shown in Figure 2. To determine β-Gal activity, the plates were first washed three times in fresh media before adding Promega β-Glo reagent according to manufacturer's instructions and monitoring luminescence using a TopCount luminometer (PerkinElmer).

### Collagen VII Read-Through Assay

AD293 cells were transformed in bulk with one of the plasmids, pJ609-R578X or pJ609-Q251X (DNA 2.0). These plasmids express human collagen VII containing the mutation R578X (TCGA to TGAG) or the mutation Q251X (CAGT to TAGT) with a C-terminal FLAG tag from the CMV promoter. Transformed cells were plated in 96-well plates from frozen stocks with G418 or PTC124 added to concentrations as shown in Figure 4. Cell supernatants were taken for analysis following 3 days of growth, and collagen VII concentration in the supernatant analysed by ELISA. ELISA plates (Nunc polysorp) were coated with anti-FLAG antibody (Abcam Ab1162) at a concentration of 2.5 µg/ml in carbonate buffer: (0.1 M NaHCO_3_/Na_2_CO_3_ pH 9.6). 50 µl of antibody solution was added to each well and the plate incubated overnight at 4°C. Between each further step, the plate was washed extensively with PBST (PBS with 0.1% Tween 20). Each incubation step was at room temperature. Tissue culture supernatant was applied and the plate incubated for 1 hour. Following washing, the plate was blocked for 1 hour with 2% skimmed milk in PBST. The plate was washed again and the wells treated with the anti-collagen VII antibody LH7.2 (hybridoma supernatant diluted 1 in 10 with 2% skimmed milk in PBST) and the plate incubated for 1 hour. Following further washing, a secondary antibody was applied to the plate (HRP-conjugated donkey anti-mouse, Jackson), diluted in PBST containing 2% skimmed milk, and the plate incubated for 1 hour. Following further washing, a detection reagent was added (Pierce TMB substrate kit) and the plate incubated until a blue colour was visually detectable. The reaction was stopped by adding an equal volume of 2 M H_2_SO_4_ and absorbance at 450 nm monitored.

### Renilla Reporter Assay

The plasmid pRL-CMV (Promega) was subjected to site-directed mutagenesis to create the read-through reporter plasmid pRL-mut4W. Codon 21 was mutated from TGG to TGA to give a W to X change. The primers used were as follows: 5′ GTC CGC AGT GGT GAG CCA GAT GTA AAC AAA TG 3′ and 5′ CAT TTG TTT ACA TCT GGC TCA CCA CTG CGG AC 3′. This was carried out using a method similar to that described for the QuikChange site-directed mutagenesis kit (Stratagene). AD293 cells were transfected with pRL-mut4W in 96-well plates from the same plasmid/transfection reagent mix. PTC124 or G418 was added 6 hours post-transfection and RLuc activity assayed 48 hours post-transfection. Cells were washed once in PBS and then lysed in 20 µl Passive Lysis buffer (Promega) with shaking for 30 minutes. RLuc activity was measured using “Stop and Glo” buffer (Promega) according to the manufacturer's instructions with a LUMIstar OPTIMA luminometer (BMG Labtech).

### K6a-YFP Reporter Assays

A wild-type K6a-YFP fusion construct was generated and subcloned into a pcDNA5/FRT vector (Invitrogen) as described previously [18]. The arginine 533 codon in K6a was replaced by a premature TGA stop codon using the QuickChange site-directed mutagenesis system (Stratagene) with the following primers: K6aTGAG.F 5′ AGT TCC AGC AGT GCA TGA GAC ATT GGG GGT GGC 3′ and K6aTGAG.R 5′ GCC CCC CCA ATG TCT CAT GCA CTG CTG GAA CT 3′. An upstream GGC and a downstream GCC flanking the premature termination codon were also replaced by GCA and GAC respectively.

AD293 cells were transiently transfected with the mutated K6a-YFP vector carrying R533X in the K6A gene and co-transfected with pEGFP-C1 (Clontech) as transfection control (pEGFP-C1 was added to the transfection reagent mix then it was aliquoted to add the individual K6a-YFP plasmids). Six hours following transfection, the medium was replaced by fresh medium with 200 µM G418 or 3 µM PTC124 with a final DMSO concentration of 1% for each. After 24 hours' incubation with compounds, the cells were harvested in PBS and the pellet was resuspended in 150 µl of extraction buffer that consisted of NuPAGE LDS Sample Buffer (Invitrogen) and NuPAGE Sample Reducing Agent (Invitrogen). Samples were analysed by electrophoresis using NuPAGE Novex 4%–12% Bis-Tris gels (Invitrogen) and transferred onto nitrocellulose membranes (Whatman). Before loading, samples were sonicated and heated for 10 minutes at 70°C. YFP expression was detected by anti-GFP monoclonal antibody (Roche) and AlexaFluor 680 F(ab′) fragment of goat anti-mouse IgG (Invitrogen) and visualised by Li-cor Imaging System using Odyssey 2.1 software. The blots were also probed with a primary antibody specific to keratin 18 (Abcam Ab31844) as a loading control; for transfection control the same anti-GFP antibody as above was used to detect GFP. All blots were scanned and analysed with the same instrument settings.

Termination efficiencies are influenced not only by the nature of the stop codon, but also the nature of the nucleotide following the nonsense codon (+1 position). Therefore, a panel of constructs was created with every possible stop codon combined with every possible +1 nucleotide using the QuickChange site-directed mutagenesis system. The primers used for the mutagenesis are as follows.

(for TGAA) K6aTGAA.F 5′ AGT TCC AGC AGT GCA TGA AAC ATT GGG GGT GGC 3′ K6aTGAA.R 5′ GCC ACC CCC AAT GTT TCA TGC ACT GCT GGA ACT 3′

(for TGAC) K6aTGAC.F 5′ AGT TCC AGC AGT GCA TGA CAC ATT GGG GGT GGC 3′ K6aTGAC.R 5′ GCC ACC CCC AAT GTG TCA TGC ACT GCT GGA ACT 3′

(for TGAT) K6aTGAT.F 5′ AGT TCC AGC AGT GCA TGA TAC ATT GGG GGT GGC 3′ K6aTGAT.R 5′ GCC ACC CCC AAT GTA TCA TGC ACT GCT GGA ACT 3′

(for TAGG) K6aTAGG.F 5′ AGT TCC AGC AGT GCA TAG GAC ATT GGG GGT GGC 3′ K6aTAGG.R 5′ GCC ACC CCC AAT GTC CTA TGC ACT GCT GGA ACT 3′

(for TAGA) K6aTAGA.F 5′ AGT TCC AGC AGT GCA TAG AAC ATT GGG GGT GGC 3′ K6aTAGA.R 5′ GCC ACC CCC AAT GTT CTA TGC ACT GCT GGA ACT 3′

(for TAGC) K6aTAGC.F 5′ AGT TCC AGC AGT GCA TAG CAC ATT GGG GGT GGC 3′ K6aTAGC.R 5′ GCC ACC CCC AAT GTG CTA TGC ACT GCT GGA ACT 3′

(for TAGT) K6aTAGT.F 5′ AGT TCC AGC AGT GCA TAG TAC ATT GGG GGT GGC 3′ K6aTAGT.R 5′ GCC ACC CCC AAT GTA CTA TGC ACT GCT GGA ACT 3′

(for TAAG) K6aTAAG.F 5′ AGT TCC AGC AGT GCA TAA GAC ATT GGG GGT GGC 3′ K6aTAAG.R 5′ GCC ACC CCC AAT GTC TTA TGC ACT GCT GGA ACT 3′

(for TAAA) K6aTAAA.F 5′ AGT TCC AGC AGT GCA TAA AAC ATT GGG GGT GGC 3′ K6aTAAA.R 5′ GCC ACC CCC AAT GTT TTA TGC ACT GCT GGA ACT 3′

(for TAAC) K6aTAAC.F 5′ AGT TCC AGC AGT GCA TAA CAC ATT GGG GGT GGC 3′ K6aTAAC.R 5′ GCC ACC CCC AAT GTG TTA TGC ACT GCT GGA ACT 3′

(for TAAT) K6aTAAT.F 5′ AGT TCC AGC AGT GCA TAA TAC ATT GGG GGT GGC 3′ K6aTAAT.R 5′ GCC ACC CCC AAT GTA TTA TGC ACT GCT GGA ACT 3′

## Supporting Information

Figure S1 **Figurative description of the various reporter assays used in the study indicating the sequence context and relative position of premature stop codons within the gene.**
(PDF)

Figure S2 **LCMS analysis of PTC124.**
(PDF)

Figure S3 **NMR analysis of PTC124.**
(PDF)

## Abbreviations

CF: cystic fibrosis
DMD: Duchenne muscular dystrophy
FLuc: Firefly luciferase
PTC: premature termination codon
RLuc: *Renilla* luciferase
