# Supplementary figures and images for: A Lack of Premature Termination Codon Read-Through Efficacy of PTC124 (Ataluren) in a Diverse Array of Reporter Assays
Source: PLoS Biol. 2013 Jun 25;11(6):e1001593. doi: 10.1371/journal.pbio.1001593 (PMC3692445; doi:10.1371/journal.pbio.1001593)

Figure S1

## Reporter construct

## Readout

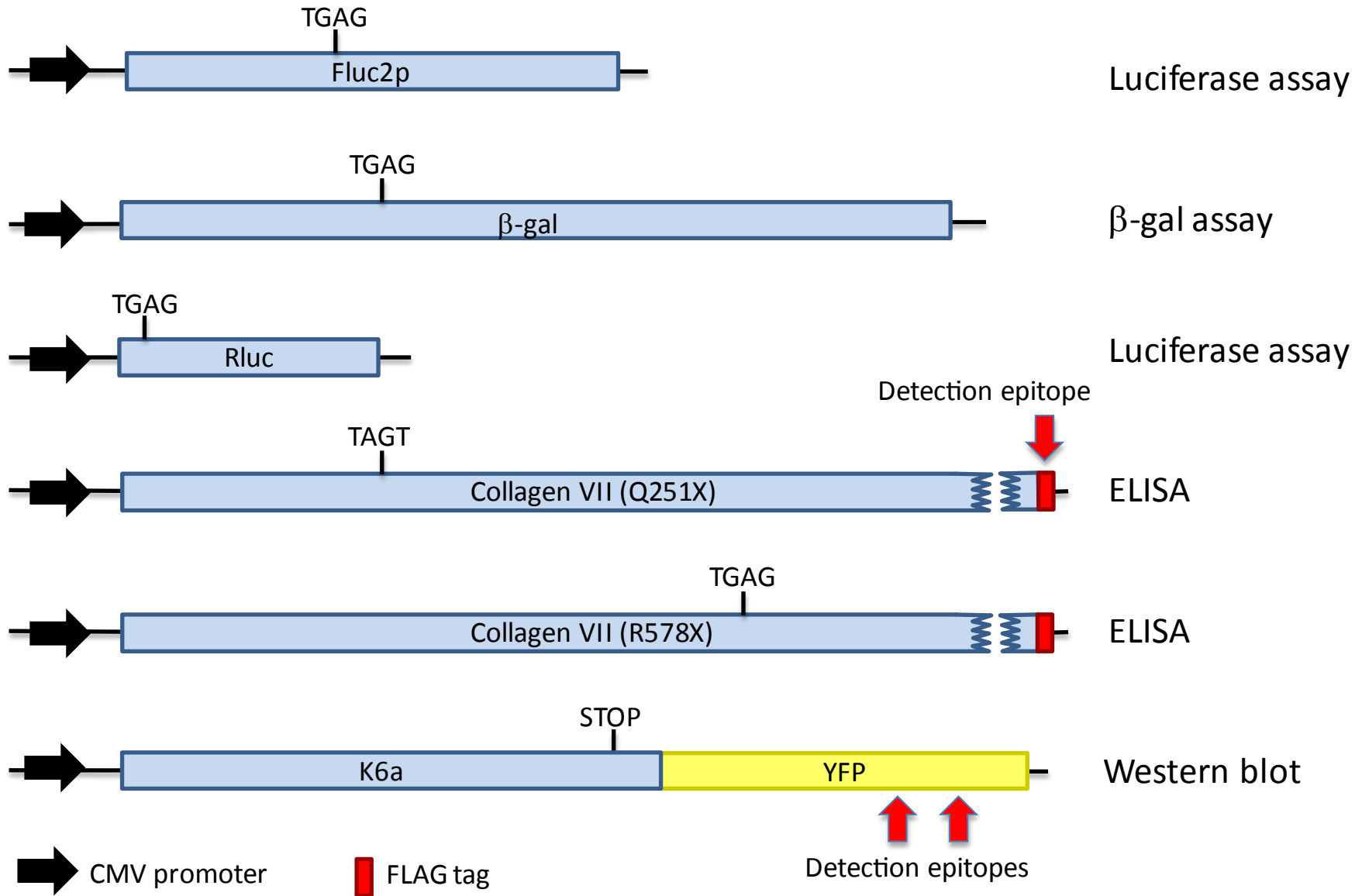

Supplement: Figure S1 — Figurative description of the various reporter assays used in the study indicating the sequence context and relative position of premature stop codons within the gene. (PDF) [file pbio.1001593.s001.pdf]
