# Supplementary material for: A Lack of Premature Termination Codon Read-Through Efficacy of PTC124 (Ataluren) in a Diverse Array of Reporter Assays
Source: PLoS Biol. 2013 Jun 25;11(6):e1001593. doi: 10.1371/journal.pbio.1001593 (PMC3692445; doi:10.1371/journal.pbio.1001593)
Supplement: Figure S2 — LCMS analysis of PTC124. (PDF) [file pbio.1001593.s002.pdf]

Figure S2

Chromatogram List Report

Analysis Info

Analysis Name	D:\Data\ian gilbert\gw\PTC-124_3_01_46163.d	Acquisition Date	10/02/2012 10:03:54
Method	vialxbridge_5-95_acetonitrile_7.5mins.m	Operator	Administrator
Sample Name	PTC-124	Instrument	micrOTOF 101
Comment			

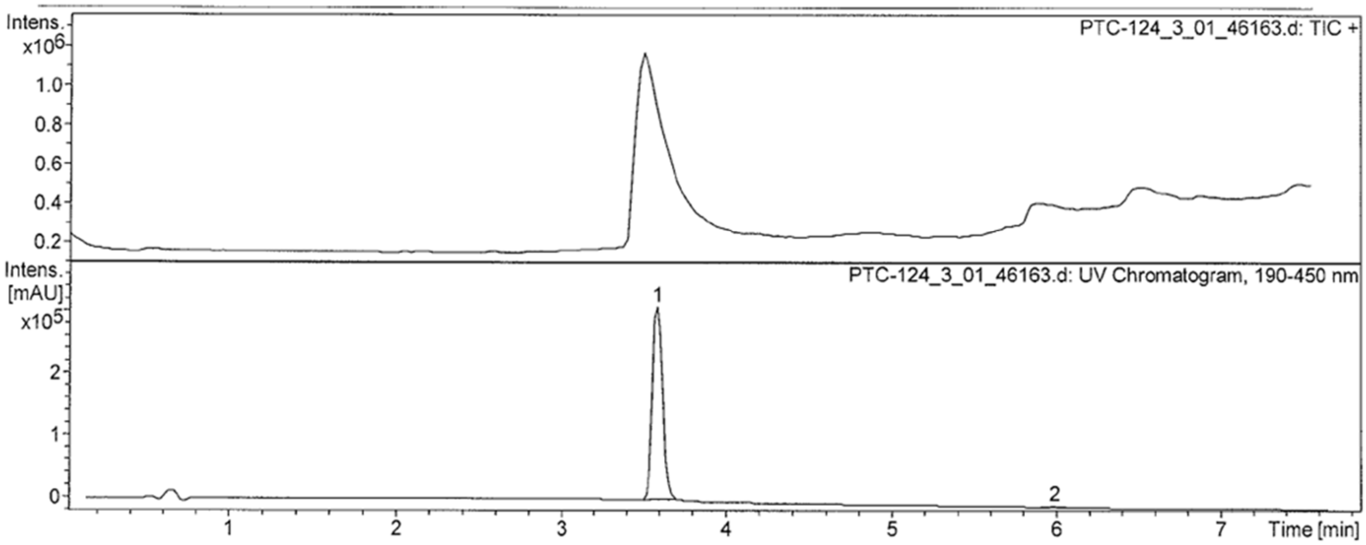

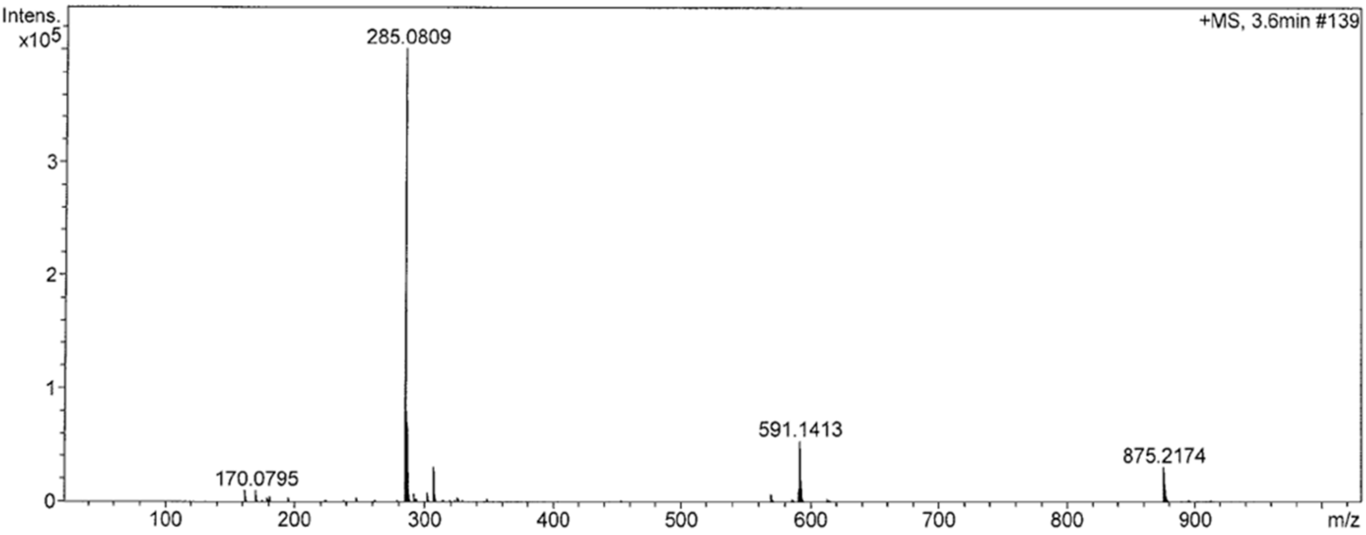

#	RT [min]	Area	Area Frac. %
1	3.6	1352578.6	99.8
2	6.0	3369.1	0.2
n.a.	3.6	n.a.	n.a.

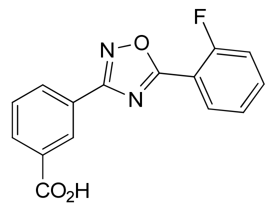

Chemical Formula: $C_{15}H_9FN_2O_3$
Molecular Weight: 284.24
