# Supplementary material for: A Lack of Premature Termination Codon Read-Through Efficacy of PTC124 (Ataluren) in a Diverse Array of Reporter Assays
Source: PLoS Biol. 2013 Jun 25;11(6):e1001593. doi: 10.1371/journal.pbio.1001593 (PMC3692445; doi:10.1371/journal.pbio.1001593)
Supplement: Figure S3 — NMR analysis of PTC124. (PDF) [file pbio.1001593.s003.pdf]

Figure S3

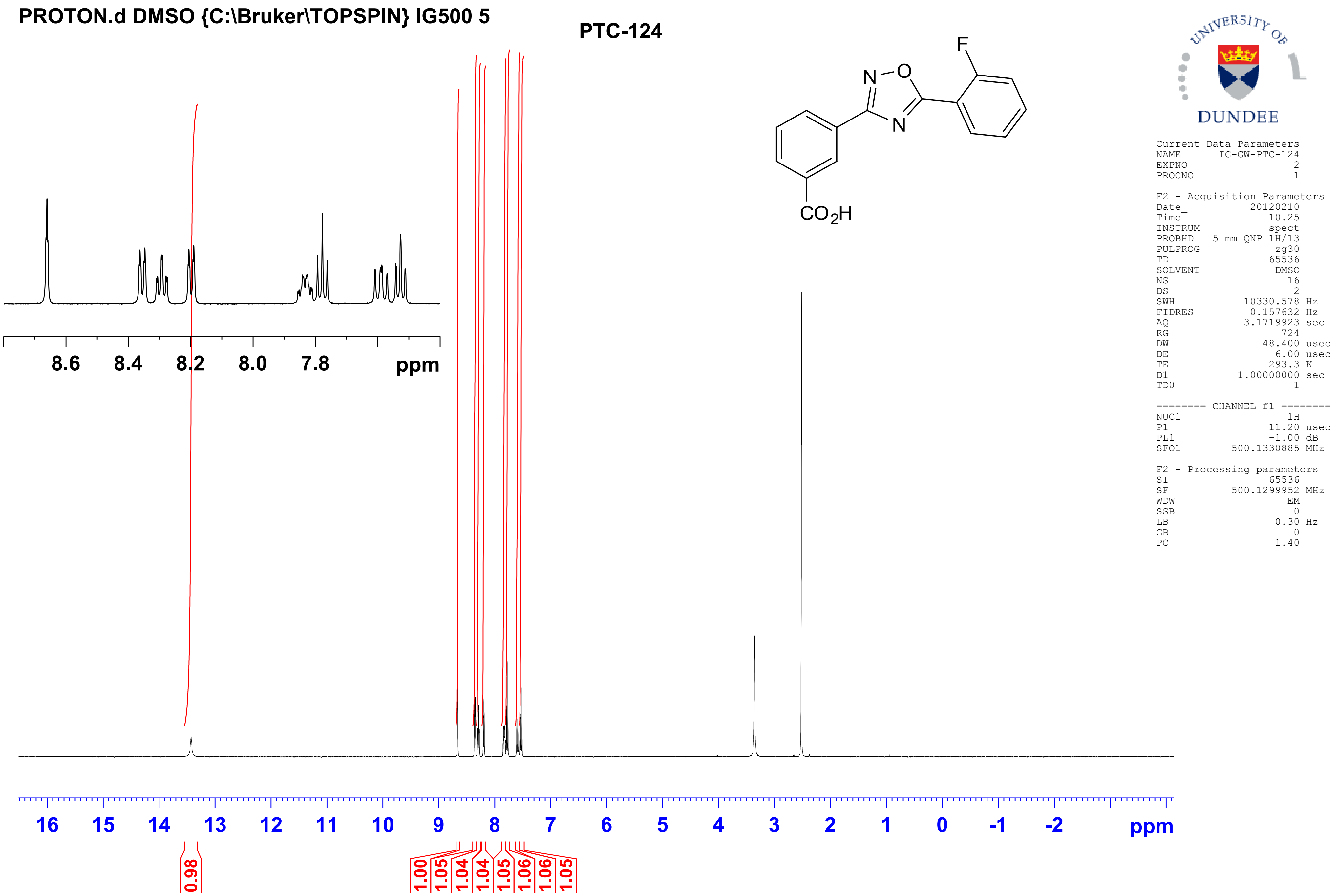
PROTON.d DMSO {C:\Bruker\TOPSPIN} IG500 5
PTC-124
F
N
O
N
CO_2H
8.6
8.4
8.2
8.0
7.8
ppm
16
15
14
13
12
11
10
9
8
7
6
5
4
3
2
1
0
-1
-2
ppm
0.98
1.00
1.05
1.04
1.04
1.05
1.06
1.06
1.05
UNIVERSITY OF
DUNDEE
Current Data Parameters
NAME IG-GW-PTC-124
EXPNO 2
PROCNO 1
F2 - Acquisition Parameters
Date_ 20120210
Time 10.25
INSTRUM spect
PROBHD 5 mm QNP 1H/13
PULPROG zg30
TD 65536
SOLVENT DMSO
NS 16
DS 2
SWH 10330.578 Hz
FIDRES 0.157632 Hz
AQ 3.1719923 sec
RG 724
DW 48.400 usec
DE 6.00 usec
TE 293.3 K
D1 1.00000000 sec
TD0 1
======== CHANNEL f1 ========
NUC1 1H
P1 11.20 usec
PL1 -1.00 dB
SFO1 500.1330885 MHz
F2 - Processing parameters
SI 65536
SF 500.1299952 MHz
WDW EM
SSB 0
LB 0.30 Hz
GB 0
PC 1.40
